# Continuous Magnetoelectric Control in Multiferroic DyMnO_3_ Films with Twin-like Domains

**DOI:** 10.1038/srep20175

**Published:** 2016-02-02

**Authors:** Chengliang Lu, Hakan Deniz, Xiang Li, Jun-Ming Liu, Sang-Wook Cheong

**Affiliations:** 1 School of Physics & Wuhan National High Magnetic Field Center, Huazhong University of Science and Technology, Wuhan 430074, China; 2 Max Planck Institute of Microstructure Physics, Weinberg 2, D-06120 Halle (Saale), Germany; 3 Laboratory of Solid State Microstructures and Innovation Center of Advanced Microstructures, Nanjing University, Nanjing 210093, China; 4 Rutgers Center for Emergent Materials and Department of Physics and Astronomy, Rutgers University, Piscataway, New Jersey, 08854, USA

## Abstract

The magnetic control of ferroelectric polarization is currently a central topic in the multiferroic researches, owing to the related gigantic magnetoelectric coupling and fascinating physics. Although a bunch of novel magnetoelectric effect have been discovered in multiferroics of magnetic origin, the manipulation of polarization was found to be fundamentally determined by the microscopic origin in a certain multiferroic phase, hindering the development of unusual magnetoelectric control. Here, we report emergent magnetoelectric control in DyMnO_3_/Nb:SrTiO_3_ (001) films showing twin-like domain structure. Our results demonstrate interesting magnetically induced partial switch of polarization due to the coexistence of polarizations along both the *a*-axis and *c*-axis enabled by the twin-like domain structure in DyMnO_3_ films, despite the polarization-switch was conventionally believed to be a one-step event in the bulk counterpart. Moreover, a continuous and periodic control of macroscopic polarization by an in-plane rotating magnetic field is evidenced in the thin films. This distinctive magnetic manipulation of polarization is the consequence of the cooperative action of the twin-like domains and the dual magnetic origin of polarization, which promises additional applications using the magnetic control of ferroelectricity.

Multiferroics of magnetic origins have become a fertile ground of understanding the coupling between magnetic and ferroelectric (FE) order parameters, which provide the possibilities of mutual controlling of electric polarization *P* (magnetization *M*) by magnetic field *H* (electric field *E*)^1^,^2^,^3^,^4^,^5^,^6^,^7^. In general, the correlation between the two ferroic orders can be built up via three mechanisms. The first one is the inverse Dzyaloshinskii-Moriya interaction (DMI) mechanism generating a polarization ***P*** ~ ***e***_*ij*_ × (***S***_*i*_ × ***S***_*j*_), where ***e***_*ij*_ denotes the unit vector connecting the neighboring spins ***S***_*i*_ and ***S***_*j*_, as long as the two spins are noncollinear^8^. The second one refers to the symmetric exchange striction which leads to a polarization ***P*** ~ ***S***_*i*_ · ***S***_*j*_^9^,^10^,^11^,^12^. The spin-dependent *p-d* hybridization which accommodates a polarization ***P*** ~ (***S***_*i*_  · ***e***_*il*_)^2^ · ***e***_*il*_, where ***e***_*il*_ is the vector connecting the transition metal and neighboring ligands^13^, is the third one. Naturally, the magnetically induced polarization can be effectively modulated by magnetic field *H* in these materials, giving rise to large magnetoelectric (ME) coupling, as first discovered in TbMnO_3_ (TMO)^14^. However, the magnetic control of polarization can be fundamentally distinct in different multiferroics because of the different microscopic origins and microstructural details. In some cases, the polarization generation can be of dual or triple magnetic origin, while in some other cases the specific domain structures may bring in additional variants to the control roadmaps.

Not only for practical applications, magnetic control of polarization is also a central topic in condensed matter physics and has been studied intensively in recent years. In particular, for multiferroics with spiral spin order (SSO), such as orthorhombic manganites *R*MnO_3_ (*R* is the rare earth element), polarization ***P*** generated *via* the inverse DMI mechanism can be completely switched *via* the *H*-driven 90° flop of the spiral spin plane. This event is commonly accompanied with a gigantic ME coupling^15^,^16^,^17^,^18^,^19^. The well-known demonstration of the second mechanism (***P*** ~ ***S***_*i*_ · ***S***_*j*_) was given in orthoferrite DyFeO_3_ where *P* can be tuned by *H*-realignment of the spins^20^,^21^. So far only a few multiferroics were found to follow the third mechanism and the generated *P* is usually small but can be smoothly rotated due to the *H*-driven rotation of ***S***_*i*_, as evidenced in Ba_2_CoGe_2_O_7_^13^. Other than this, quite a few of multiferroics have been demonstrated to accommodate more than one mechanism, i.e. dual or triple magnetic origin of polarization generation. In this case, magnetic control of polarization becomes an issue with implication of additional ingredient since every mechanism does offer its specific behaviors, making the underlying physics affluent. Actually, this issue would be of high interest and practical significance, since the dual or triple magnetic origin, if properly utilized, may open additional possibilities for distinctive ME control roadmaps.

One of the best objects for settling this issue is orthorhombic DyMnO_3_ (DMO) in which the polarization is of dual magnetic nature^22^,^23^,^24^, as illustrated in Fig. 1(a) for the lattice and spin structures in a certain temperature (*T*) range. A polarization *P* along the *c*-axis (*P*_Mn_) is generated *via* the first mechanism associated with the *bc*-plane Mn SSO state^25^. In addition, the strong Dy-Mn coupling can drive the Dy spins to align coherently with the Mn SSO state, thus contributing a polarization along the *c*-axis too (*P*_Dy_), *via* the second mechanism, i.e. the symmetric exchange striction between the Dy and Mn spins (*P*_Dy_ ~ *S*_Dy_ · *S*_Mn_)^22^,^23^,^24^. A detailed description of this consequence can be found in the Supplementary materials, Figure S1. It is seen that the *bc*-plane Dy spins align in parallel or antiparallel to the neighboring Mn spins, and the total polarization of dual magnetic origin becomes *P*_*c*_ = *P*_Mn_ + *P*_Dy_, allowing large polarization and gigantic ME control. This effect has been revealed in bulk DMO and confirmed in DMO thin films deposited on (001) SrTiO_3_ substrates^26^. Besides, the Dy-Mn coupling was revealed to modify the multiferroic phase diagram of DMO drastically^27^.

While the dual magnetic origin motivated us to explore an emergent manipulation of *P* in DMO, possible undiscovered ME control stemming from domain engineering would further inspire such a motivation^28^,^29^,^30^. For bulk DMO, the polarization *P* can be switched from the *c*-axis to the *a*-axis (*P*_*c*_ → *P*_*a*_) by field *H* along the *b*-axis or *a*-axis (Fig. 1(b)), as the consequence of the Mn spin spiral flop from the *bc*-plane to the *ab*-plane^25^. The critical fields for the two flops are denoted as *H*_*c1*_ and *H*_*c2*_. The *P*_*c*_ → *P*_*a*_ switching can be triggered more easily by the *H*//*b*-axis than that along the *a*-axis, and the difference (*H*_*c2*_ − *H*_*c1*_) can be as big as ~6.0 T at *T* = 5 K^25^. Apart from the above physics, a major issue goes to DMO thin films epitaxially grown on proper substrates. Indeed, several recent studies on *R*MnO_3_ thin films on (001) SrTiO_3_ (STO) revealed general twin-like domain structures^26^,^29^,^31^,^32^,^33^,^34^. As revealed^26^, some of the well-known multiferroic properties of bulk DMO have been identified in epitaxial DMO films. The twin-like domain structure allows sufficient space to explore additional strategies towards the magnetic control of polarization. For example, one can subtly make use of the distinct *P*-switch sequences upon *H* applied along the *b*-axis or *a*-axis, respectively. The two types of domains in the twin-like domain structure, i.e. *α*-domains and *β*-domains, can have concurrent or distinctly different *P*-switching events, *i.e.* emergent phenomena absent in bulk DMO.

In the present work, we will explore these phenomena in high quality DMO thin films by measuring the response of dielectric permittivity *ε* and polarization *P* to *H* in different spatial geometries. The two distinct *P*-switching sequences enabled by the twin-like domain structure will be demonstrated. In particular, we have observed an interesting smooth and periodic modulation of macroscopic *P* by in-plane rotating *H*, ascribed to the twin-like domain structure and the dual magnetic origin of polarization.

## Results

### Twin-like domain structure in DMO films

**Figure 2:**
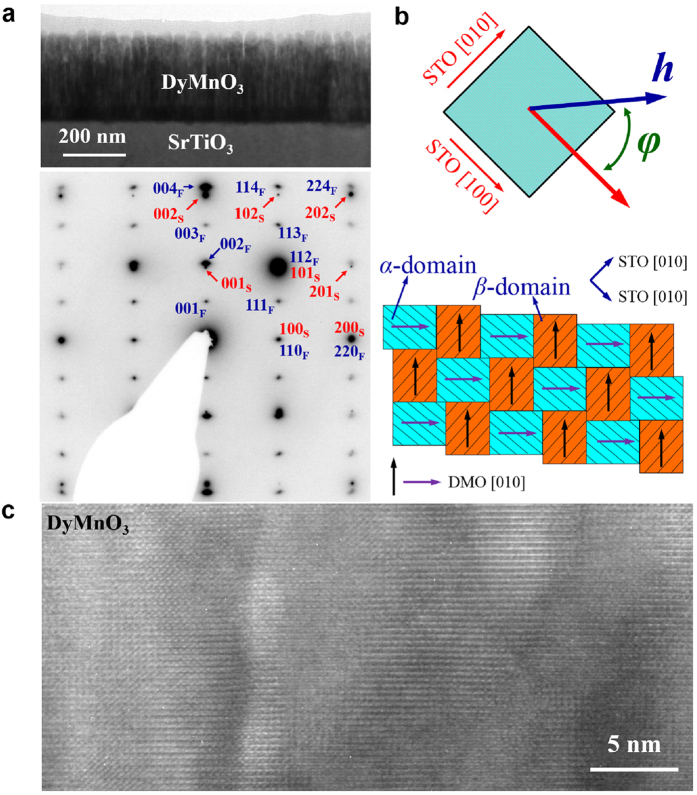
TEM observations of microstructure in a DMO thin film. (**a**) Top: cross-section transmission electron microscopy of the film of ~270 nm in thickness. Bottom: corresponding electron diffraction pattern along [010]_S_. The subscript ‘S’ and ‘F’ denote substrate and film, respectively. (**b**) Top: definition of angle *φ*, which is the angle between the applied magnetic field and [100] of the SrTiO_3_ substrate. Bottom: Sketch of the twin-like domain structure composed of *α*- and *β*-domains, in which arrows indicate the local domain [010] directions of the film. (**c**) High resolution transmission electron microscopy image of the film.

The cross-section transmission electron microscopy (TEM) image in Fig. 2(a) shows the column structure of one sample, which represents the typical characteristic of multiferroic *R*MnO_3_ thin films on (001) STO, arising from the twin-like structure due to its most efficient release of the in-plane strain, consistent with our previous reciprocal-space-mapping data and twin-like diffraction spots^26^. The corresponding diffraction pattern in Fig. 2(a) plus the clear lattice planes revealed by the high resolution TEM image (Fig. 2(c)) indicate the single crystal nature and high quality of the film. Additional plane view TEM provides more evidence of the twin-like domain structure, shown in the Supplementary materials, Figure S2. The twin-like structure consisting of alternatively stacked in-plane *α*- and *β*-domains is drawn in Fig. 2(b) where *φ* is the angle between in-plane *H* and STO [100]/DMO <110> direction and the rectangles show the domains with arrows indicating the [010] directions (i.e. the *b*-axis) of the local domains. At *φ* = 0, field *H* aligns along the diagonal directions of both the *α*- and *β*-domains, and the *bc*-spiral planes of the two types of domains are expected to flop simultaneously onto the *ab*-plane under sufficient *H* (*H* > √2*H*_*c1*_). At *φ* = 45°, field *H* is in parallel to the *b*-axis of the *α*-domains but perpendicular to that of the *β*-domains, possibly leading to the flop of the *bc*-spiral spin plane of the *α*-domains at *H* > *H*_*c1*_ and then to the flop of the *bc*-spiral spin plane of the *β*-domains at *H* > *H*_*c2*_.

**Figure 3:**
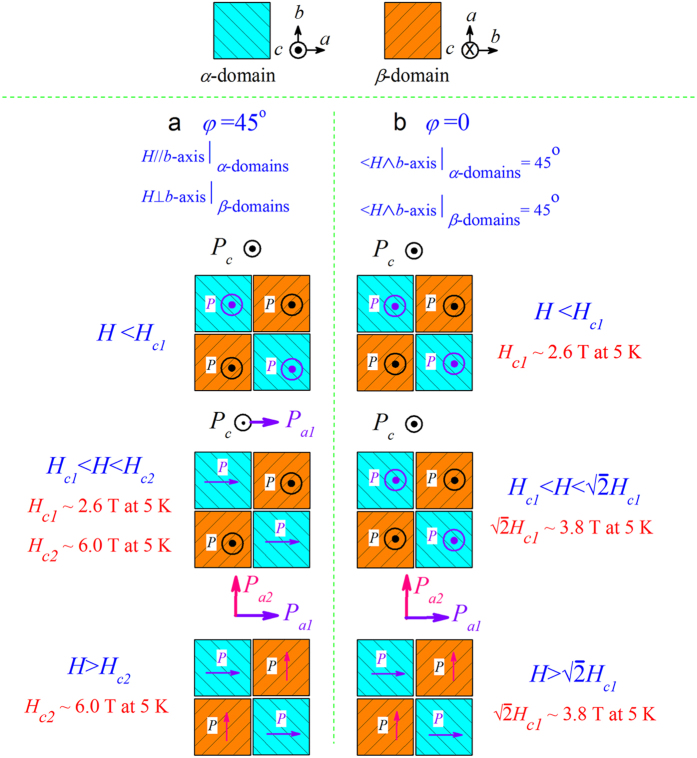
Sketch of concurrent and distinct polarization switching events as *H* is applied at (**a**) *φ* = 45° and (**b**) *φ* = 0 in DMO thin films. Macroscopic polarization, including *P*_*c*_, *P*_*a1*_ (due to *P*-switching of *α*-domains), and *P*_*a2*_ (due to *P*-switching of *β*-domains), of each polarization switching event are also illustrated.

Based on the twin-like domain structure, one can propose the pathway by which the two types of domains are switched. The two cases at *φ* = 45° and *φ* = 0 as examples are sketched in Fig. 3(a,b) at low *T*, where the values of *H*_*c1*_ and *H*_*c2*_ are taken from our measurements at *T* = 5 K. Given the initial state that all the domains have their *P* along the *c*-axis (*P*_*c*_), we look at the *P*-switching of the two cases. At *φ* = 45°, the *P*-switching of the *α*-domains occurs at *H* > *H*_*c1*_ but the *β*-domains remains non-flopped until *H* > *H*_*c2*_ ≫ *H*_*c1*_. This implies that the remnant *P*_*c*_ in *H*_*c1*_ < *H* < *H*_*c2*_ would be roughly a half of that at the initial state. Similar analysis can be done for *φ* = 0 but the consequence is different. Both the *α*- and *β*-domains have the *P*_*c*_ → *P*_*a*_ switching only at *H* > √2*H*_*c1*_ when the *H*-components along the *b*-axis of both *α*- and *β*-domains are higher than *H*_*c1*_.

### Magnetic control of polarization

**Figure 4:**
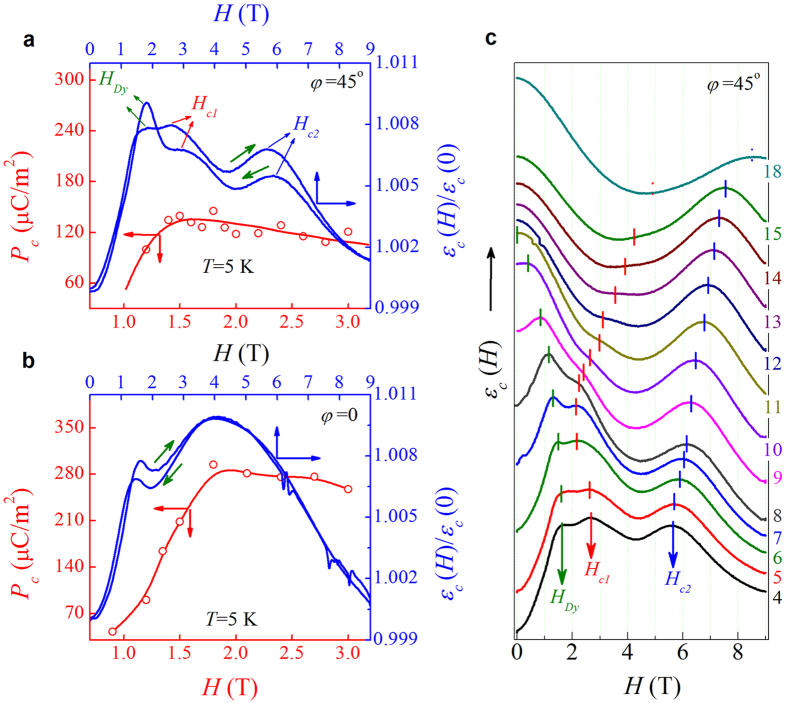
Magnetoelectric coupling in DyMnO_3_ thin film. Measured *P*_*c*_ and *ε*_*c*_ as a function of *H* applied at (**a**) *φ* = 45° and (**b**) *φ* = 0 at *T* = 5 K. (**c**) Magneto-dielectric data measured at various *T* with a step of 1 K from 4 K to 15 K. The short bars indicate the fields *H*_Dy_, *H*_*c1*_, and *H*_*c2*_.

Given the proposed scheme in Fig. 3, one can now investigate in details the *P*-switching induced by *H*. It is noted that the Mn SSO state and thus the induced Dy incommensurate (ICM) order are robust against magnetic fields beyond a threshold of ~10.0 T, although the spiral spin plane will flop at a field far below this threshold^25^. We first look at the *ε*_*c*_(*H*) and *P*_*c*_(*H*) at *φ* = 45° and *T* = 5 K below the Dy’s spin ordering temperature *T*_Dy_ ~ 9 K, as shown in Fig. 4(a), while the *T*-dependences of *ε*_*c*_ and *P*_*c*_ can be found in the Supplementary materials, Figure S3. The *ε*_*c*_*-H* curves in the *H*-increasing and decreasing cycle show some difference but their qualitative features are similar. The first dielectric peak around *H* = *H*_Dy_ ~ 1.0 to 1.5 T is due to the variation of *P*_Dy_ associated with the commensurate (CM) ↔ ICM transition of Dy spins. The second peak around *H* = *H*_*c1*_ ~ 2.6 T features the *P*_*c*_ ↔ *P*_*a*_ switching of the *α*-domains. The third peak at *H* = *H*_*c2*_ ~ 6.0 T reflects the *P*_*c*_ ↔ *P*_*a*_ switching of the *β*-domains. The *P*_*c*_(*H*) curve obtained by the positive-up-negative-down (PUND) shows corresponding fast increase around *H*_Dy_, and then decreases gradually with further increasing *H*. Since the *P*_*c*_(*H*) was measured using PUND at a series of selected magnetic fields, it is indeed not as sensitive as the dielectric response *ε*_*c*_(*H*) obtained by sweeping the magnetic field in a continuous manner in detecting *P*-switching events.

For *φ* = 0, the measured *ε*_*c*_(*H*) and *P*_*c*_(*H*) data at *T* = 5 K are shown in Fig. 4(b). Distinctly different from the case at *φ* = 45°, here we only observe two peaks. The first peak at *H*_Dy_ ~ 1.5 T is similar to the above case. The second peak appears at *H* ~ 4.0 T instead of *H*_*c1*_ ~ 2.6 T. The reason can be easily understood from the model in Fig. 3. For *φ* = 0, the *b*-axes of all the domains are ~45° inclined from the *H* direction. The Mn/Dy spiral spin planes can’t be flopped onto the *ab*-plane until *H* ~ 4.0 T ≥ √2*H*_*c1*_. In this case, the anomaly at √2*H*_*c2*_ is no longer available. Similarly, the measured *P*_*c*_(*H*) reaches a maximum at *H* ~ 1.8 T. In addition, it is easily understood that the measured *P*_*c*_(*H*) at *H* > *H*_*c1*_ for *φ* = 45° is much smaller than that for *φ* = 0. Owing to the high sensitivity of dielectric permittivity, we measure a series of *ε*_*c*_(*H*) curves at various *T* in the *H*-increasing sequence to evaluate the *H*_Dy_, *H*_*c1*_, and *H*_*c2*_ (if any). For *φ* = 45°, the data at *T* = 4 K to 18 K are plotted in Fig. 4(c), where the numbers on the right border are temperatures and the *H*_Dy_, *H*_*c1*_, and *H*_*c2*_ are marked. As expected, the *H*_Dy_ shifts gradually towards the low-*H* side with increasing *T* until *T*_Dy_ ~ 9 K, beyond which no more Dy CM order is sustained. The *H*_*c1*_(*T*) first shifts towards the low-*H* side but turns to the high-*H* side as *T* ~ *T*_Dy_, an unusual behavior. The *H*_*c2*_(*T*) dependence is trivial by gradual increasing of *H*_*c2*_ over the whole *T*-range here.

To explain the dependences *H*_*c1*_(*T*) and *H*_*c2*_(*T*), one may discuss the stability of the *bc*-plane Mn SSO state against *H*. An increasing of *H* beyond *H*_Dy_(*T*) drives the CM-ICM transition of Dy spin order *via* the Dy-Mn coupling. This transition is compensated by the stability penalty of the Mn SSO state. As shown in Figure S3(b), *P*_Dy_ reaches the maximum at *T*_Dy_, which would impact the *bc*-plane Mn-SSO most seriously, leading to the smallest critical field *H*_*c1*_ around *T*_Dy_. At high field region, *P*_Dy_ has already been fully recovered, and will not be enhanced by *H* anymore, giving rise to a monotonous variation of *H*_*c2*_(*T*).

### Ferroelectric phase diagram

**Figure 5:**
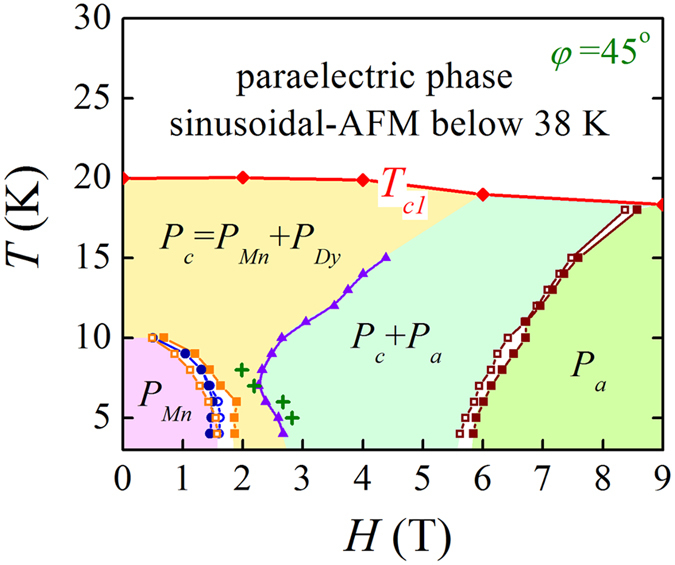
Multiferroic phase diagram of the DMO thin films with *H* at *φ* = 45°. The values of *T*_*c*_ were obtained from the *ε*_*c*_(*T*) data. Open and closed symbols denote the data obtained with increasing *H* and decreasing *H*, respectively. For a comparison, the critical fields of anomalous transitions with *H* applied at *φ* = 0 are also shown as circles (increasing *H*) and dots (decreasing *H*) in the phase diagram. The olive crosses denote the effective field *H* · cos45° along the *a-* or *b*-axis.

Based on the above discussion, we can construct the FE phase diagram in the (*H*, *T*) plane for the DMO thin films. The phase diagram for *φ* = 45° is presented in Fig. 5, where the different sets of dots are from the dielectric data in the cooling and warming sequences, respectively. The phase diagram for *φ* = 0 is qualitatively similar while the phase borders may be quantitatively different. The diagram is divided into five regions. The region at *T* > *T*_*c1*_ is occupied by the paraelectric phase. At 10 K < *T* < 20 K there appear in order the FE phase with *P*//*c*-axis (*P*_*c*_), the FE phase with mixed components *P*//*c*-axis and *P*//*a*-axis (*P*_*c*_ + *P*_*a*_), and the FE phase with *P*//*a*-axis (*P*_*a*_), with increasing *H*. Below *T* = 10 K, the increasing *H* drives the transitions from the FE phase with *P*//*c*-axis (*P*_*c*_ = *P*_Mn_), to the FE phase with *P*//*c*-axis (*P*_*c*_ = *P*_Mn_ + *P*_Dy_), then to the FE phase with mixed components *P*//*c*-axis and *P*//*a*-axis (*P*_*c*_ + *P*_*a*_), and eventually to the FE phase with *P*//*a*-axis (*P*_*a*_).

It is seen that the phase diagram of the DMO thin films shows almost identical features to an integration of the two phase diagrams for bulk DMO, given *H*//*a*-axis and *H//b*-axis respectively^25^. However, the *H*-driven *P*_*c*_ ↔ *P*_*a*_ transition is not as sharp as that in bulk DMO. Instead, a broad region with coexisting *P*_*a*_ and *P*_*c*_ domains is identified in the phase diagram, primarily due to the in-plane twin-like domain structure of the DMO thin films.

### Continuous control of macroscopic polarization

**Figure 6:**
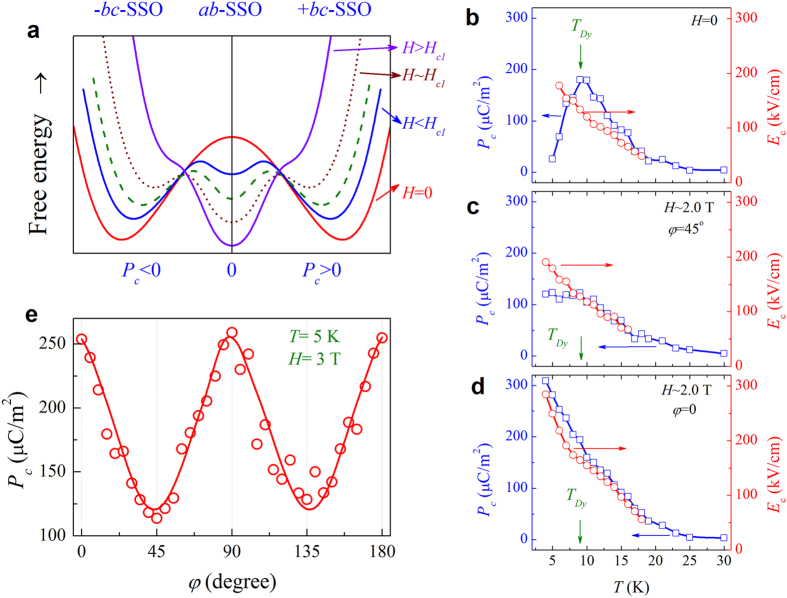
(**a**) Energy diagram on ferroelectric switching under various magnetic fields. Measured *P*_*c*_(*T*) and *E*_c_(*T*) data with *H* = 0 (**b**), *H* = 2.0 T at *φ* = 45° (**c**), and *H* = 2.0 T at *φ* = 0 (**d**). (**e**) Angular dependence of *P*_*c*_ measured under *H* = 3 T at *T* = 5 K.

While the central issue of multiferroic applications is the electric and magnetic controls of *P* and *M*, it is more significant to investigate the FE hysteresis under modulation of magnetic field. In the framework of Landau theory on ferroelectricity, the free energy *F*(*P*_*c*_, *H*) can be schematically drawn in Fig. 6(a) for a guide of eyes. The *P*_*c*_ = 0 state corresponds to the *ab*-plane SSO state where the *P*_*a*_ reaches the maximum. Intentionally, one expects that a highly sensitive modulation of *P* by *H* should be reachable around *H* ~ *H*_*c1*_ to √2*H*_*c1*_, at which there are three comparable free energy minimal states with relatively low barriers between them, so that the inter-state transitions are kinetically easy. In our experiments, we choose *H* ≥ 2.0 T and *T* ~ 5–10 K, noting that *H*_*c1*_ ~ 2.0 T in this *T*-range. A series of *P*_*c*_-*E* loops at various *T* in the cooling sequence for both *φ* = 45° and *φ* = 0, using the PUND method, have been cautiously measured. The saturated *P*_*c*_(*T*) and coercive field *E*_*c*_(*T*) are evaluated from these loops, and summarized in Fig. 6(b–d), respectively.

In general sense, the data in Fig. 6 are consistent with the model predictions given in Fig. 3. For *H* = 0, the measured *P*_*c*_ increases rapidly with decreasing *T* down to *T*_Dy_, and then falls down due to the disappearance of *P*_Dy_. The measured *E*_*c*_ increases with decreasing *T* too, noting that no reliable *E*_*c*_ values can be extracted for the loops of very small *P*_*c*_. The situation for *H* ~ 2.0 T at *φ* = 45° is different, as shown in Fig. 6(c). The measured *P*_*c*_ does increase too with decreasing *T* down to *T*_Dy_, below which *P*_*c*_ remains roughly unchanged because of the recovery of *P*_Dy_. The measured *E*_*c*_(*T*) curve roughly coincides with that for *H* = 0. Here, the *P*_*c*_ at *T* > *T*_Dy_ are smaller than that for *H* = 0, due to the *H* ~ 2.0 T driven *P*_*c*_ → *P*_*a*_ switching of the *α*-domains. For the case of *H* ~ 2.0 T at *φ* = 0, as shown in Fig. 6(d), noting *H* ~ 2.0 T < √2*H*_*c1*_, insufficient for driving *P*_*c*_ → *P*_*a*_ switching in any domain, confirmed by the overlapping *P*_*c*_(*T*) curves in Fig. 6(b,d) at *T* > *T*_Dy_. The further enhancement of the *P*_*c*_ below *T*_Dy_ is ascribed to the recovery of *P*_Dy_.

To this final stage, we are in a position to track the continuous *H*-control of macroscopic *P*_*c*_ enabled by the domain engineering for the present thin films. The in-plane *H* ~ 3.0 T, which is higher than *H*_*c1*_ and lower than √2*H*_*c1*_, is rotated, triggering the *P*_*c*_ ↔ *P*_*a*_ switching of the *α*-domains and *β*-domains alternatively, so that the measured *P*_*c*_(*φ*) oscillates with the two-fold rotation symmetry. One set of *P*_*c*_(*φ*) data are plotted in Fig. 6(e). It is seen that the minimal and maximal are ~125 μC/m^2^ and ~250 μC/m^2^, consistent with the model prediction in Fig. 3. More interesting is the continuous and periodic modulation of macroscopic *P*_*c*_. This modulation is highly robust and can be well reproduced upon different swamping frequencies of the electric field. So far no such continuous modulation of polarization has been reported in either bulk DMO or other SSO multiferroics^3^,^25^. One exception, to our best knowledge, is for Eu_0.55_Y_0.45_MnO_3_ where the rotation of a newly developed conical spin structure from an *ab*-plane spiral phase was observed^16^, driven by the *H* perpendicular to the spin propagation vector. In the present work, the continuous response of the *P*_*c*_ to in-plane rotating *H* is essentially determined by the in-plane twin-like domain structure of the DMO thin films, while the *P*_Dy_ generation in DMO also makes contributions.

### Remarks

The central feature of the DMO thin films is the in-plane twin-like domain structure which determines the emergent behaviors observed here. These behaviors may allow additional functionalities, such as the continuous modulation of macroscopic polarization. Such a twin-like structure can be easily realized in many strain-sensitive materials, stimulating us to add some remarks on possible phenomena beyond those observed here. The twin-like structure may add another spatial scale/symmetry besides the lattice level scale/symmetry and macroscopic level scale/domain pattern. For the DMO thin films, the polarization generation mechanisms are on the lattice scale and the twin-like structure adds the nano-scale *α*- and *β*-domains which show the two-fold symmetry in terms of the *P* response to in-plane *H*. Because DMO has a number of companions like other *R*MnO_3_ multiferroics of the SSO state, the present work suggests similar consequences in these systems, deserving for explorations.

Beyond these in multiferroics, the impact of such an additional scale has been demonstrated to be substantial in other materials on other properties. For examples, in metals where dislocations are responsible for deformation, the nano-scale twin-like structure may bring super-plasticity and ductility, as shown in copper^35^. The twin-like structure may even enable the micro-hardness of some materials higher than diamond^36^. These are two cases where the twin-like structure exhibits super and unexpected functionalities.

## Methods

The DMO thin films were epitaxially grown on conductive Nb-doped (001) STO substrates using pulsed laser deposition method and details of the microstructural and electrical characterizations were reported earlier^26^. The epitaxial relationships are DMO (001)//STO (001) and DMO [100]/[010]//STO [110], seen in Fig. 3(b). We performed additional cross-section and plane view transmission electron microscopy and electron diffraction studies using the Philips CM20T microscope at 200 kV and high resolution TEM using the JEOL JEM-4010 microscope, in order to check the structural details of the epitaxial films.

Based on the standard capacitor structure with Au as the top electrode, the FE hysteresis loops were measured using the PUND pulse train technique (TF Analyzer 2000 from AixACCT Co) with a mode frequency of 100 Hz at various *T* during the cooling and warming processes. As the complimentary, the pyroelectric current method was also used for probing the remnant polarization under zero electric field^24^. Here, the measured polarization is the *P*_*c*_ in response to the out-of-plane electric field *E*. The out-of-plane dielectric permittivity *ε*_*c*_ (real part) was measured as a function of both *T* and *H* using the LCR meter. For the angular dependent ME coupling, *H* was aligned within the *ab*-plane. The cryogenic and magnetic field environment was provided by a Physical Properties Measurement System (PPMS, Quantum Design Inc.).

## Additional Information

**How to cite this article**: Lu, C. *et al.* Continuous Magnetoelectric Control in Multiferroic DyMnO_3_ Films with Twin-like Domains. *Sci. Rep.* **6**, 20175; doi: 10.1038/srep20175 (2016).

## Supplementary Material

Supplementary Information

## Figures and Tables

**Figure 1:**
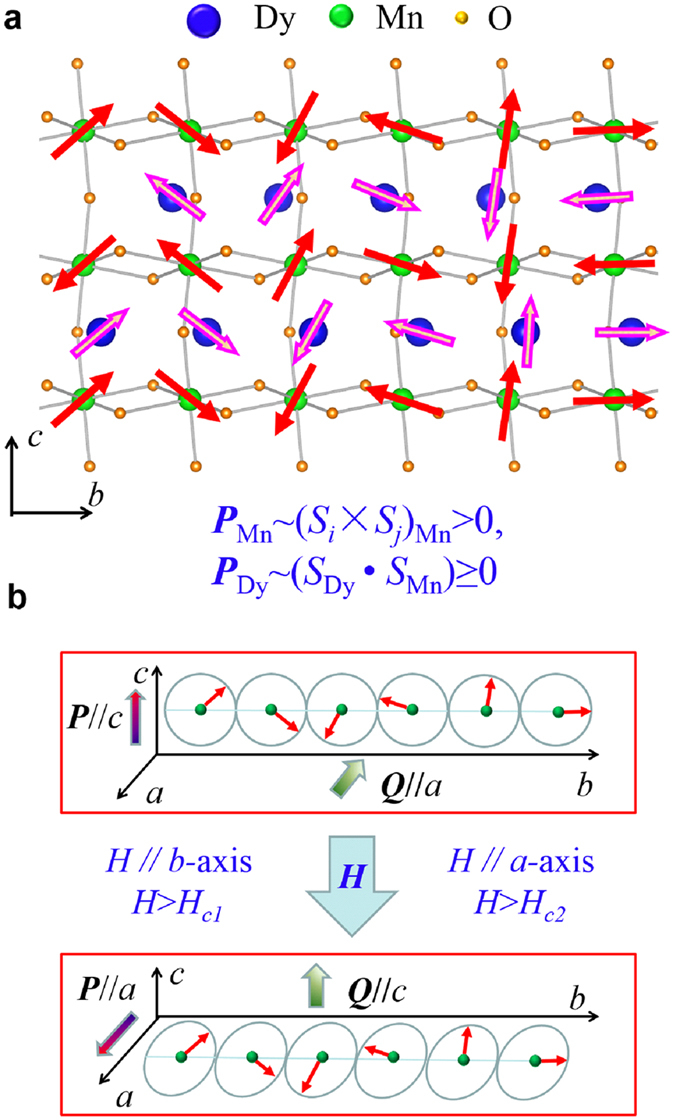
Dual multiferroicity and polarization switching in DMO. (**a**) Sketch of the coherence of magnetic configuration for Dy and Mn moments. In this picture, there are two components of the magnetically driven ferroelectric state. One comes from the spiral-spin-ordered phase of Mn moments, and the other one originates from the exchange striction between Mn and Dy spins due to their coherent configurations. (**b**) Ferroelectric polarization switching process due to the magnetic field induced 90° rotation of the spin spiral-plane. Here ***Q*** = ***S***_*i*_ × ***S***_*j*_ is the spin chirality.
