# Supplementary material for: Continuous Magnetoelectric Control in Multiferroic DyMnO3 Films with Twin-like Domains
Source: Sci Rep. 2016 Feb 2;6:20175. doi: 10.1038/srep20175 (PMC4735850; doi:10.1038/srep20175)
Supplement: Supplementary Information [file srep20175-s1.pdf]

**SUPPLEMENTARY INFORMATION**

# Continuous Magnetoelectric Control in Multiferroic $DyMnO_3$ Films with Twin-like Domains

Chengliang Lu[1,2], Hakan Deniz[2], Xiang Li[3], Jun-Ming Liu[3], and Sang-Wook Cheong[4,3]

[1]*School of Physics & Wuhan National High Magnetic Field Center, Huazhong University of Science and Technology, Wuhan 430074, China*

[2]*Max Planck Institute of Microstructure Physics, Weinberg 2, D-06120 Halle (Saale), Germany*

[3]*Laboratory of Solid State Microstructures and Innovation Center of Advanced Microstructures, Nanjing University, Nanjing 210093, China*

[4]*Rutgers Center for Emergent Materials and Department of Physics and Astronomy, Rutgers University, Piscataway, New Jersey, 08854, USA*

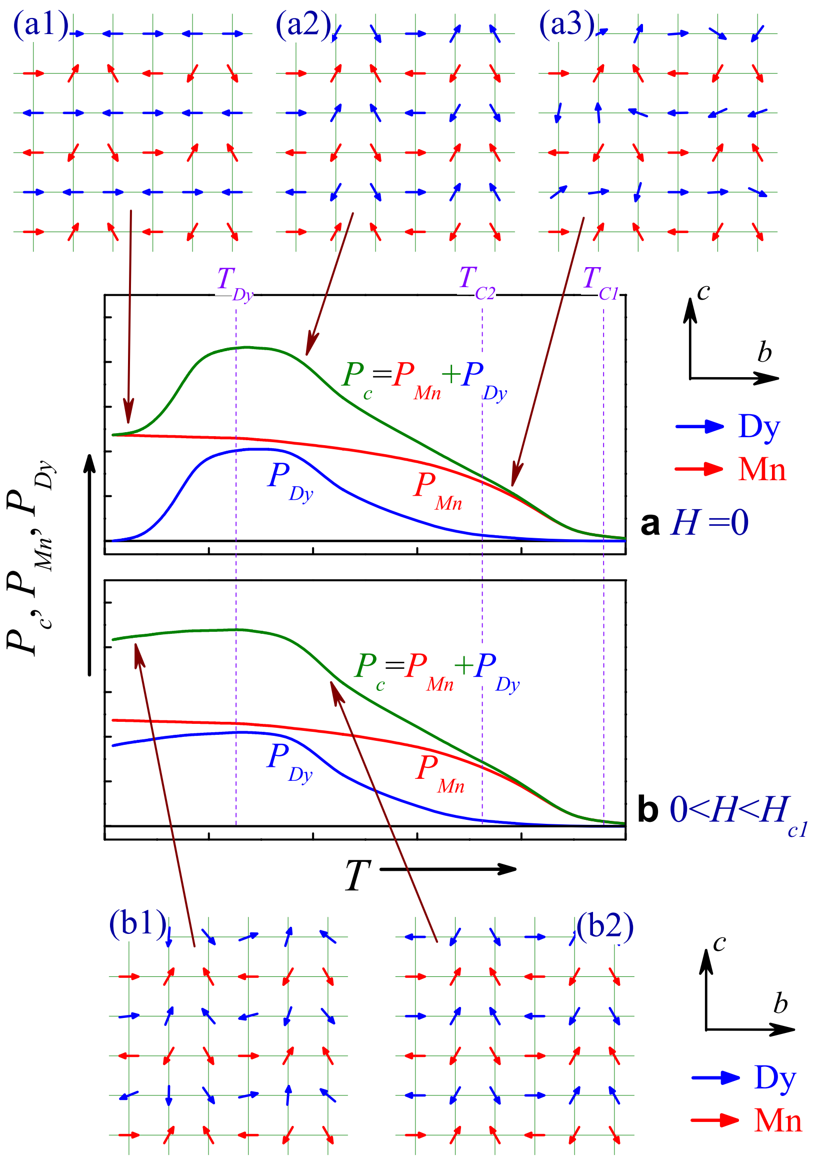


**Figure S1**. Sketch of dual multiferroicity in $DyMnO_3$, including the polarization arising from spiral spin order of Mn ($P_{\mathrm{Mn}}$) and Dy-Mn spin interaction ($P_{\mathrm{Dy}}$). Temperature dependence of the multiple-component polarization **(a)** under $H$=0, and **(b)** under $0<H<H_{c1}$. Various magnetic configurations are schematically shown in (a1)-(a3) and (b1)-(b2).

The two components $P_{\mathrm{Mn}}$ and $P_{\mathrm{Dy}}$ have different $T$-dependences due to the evolution of spin structures, and a thumbnail sketch is given in Figure S1(a) for guide of eyes. Upon cooling from high-$T$ paramagnetic state, $DyMnO_3$ enters a sinusoidal antiferromagnetic (AFM) phase at $T_{\mathrm{N}}$~39 K, and then the Mn SSO state with incommensurate (ICM) propagation vector emerges at $T_{C1}$~20 K, below which $P_{\mathrm{Mn}}$ ensues via the first mechanism (Figure S1(a3)). Simultaneously, a coherent alignment of the Dy spins with the Mn spins

develops at $T_{C2}$≤$T_{C1}$, generating $P_{\mathrm{Dy}}$ via the second mechanism (Figure S1(a2)). More than these, further cooling to $T$=$T_{\mathrm{Dy}}$ (<10 K) causes an ICM to commensurate (CM) transition of the Dy spins, which gradually destructs the Dy-Mn spin coherency and gradually eliminates the $P_{\mathrm{Dy}}$ [20], as shown in Figure S1(a1). Below $T_{\mathrm{Dy}}$, $P_{\mathrm{Mn}}$ arising purely from the Mn-SSO just shows very weak dependence on $T$. Importantly, such a weak $T$ dependence of $P_{\mathrm{Mn}}$ related to Mn-SSO with a locked propagation vector below $T_{\mathrm{C}}$, is a common feature in multiferroics with SSO, such as the cases in $TbMnO_3$ [12] and $Eu_{1-x}Y_xMnO_3$ [14]. This allows us to estimate the $P_{\mathrm{Mn}}$ *vs* $T$ in DMO. Then, $P_{\mathrm{Dy}}$-$T$ can be obtained after subtracting $P_{\mathrm{Mn}}$-$T$ from the measured $P_c$-$T$. A detailed discussion about this issue can be found in a previous mini-review paper [35]. The qualitative $T$-dependences of the $P_{\mathrm{Mn}}$, $P_{\mathrm{Dy}}$, and then $P_c$ are plotted in Figure S1(a). As shown in Figure S1(b), a field of ~$H_{c1}$ is sufficient to destroy the Dy CM order below $T_{\mathrm{Dy}}$ and regain the coherency of the Dy spins with the Mn SSO state. The consequence of such a $H$-driven recovery of the Dy spin order is the gradual re-generation of $P_{\mathrm{Dy}} \sim \boldsymbol{S}_{\mathrm{Dy}} \cdot \boldsymbol{S}_{\mathrm{Mn}}$, making the distinct difference in $P_c(T)$ between $H$=0 and $H$>0. It is argued that this coherency can sustain beyond the flops of the Mn spin spiral from the *bc*-plane to the *ac*-plane.

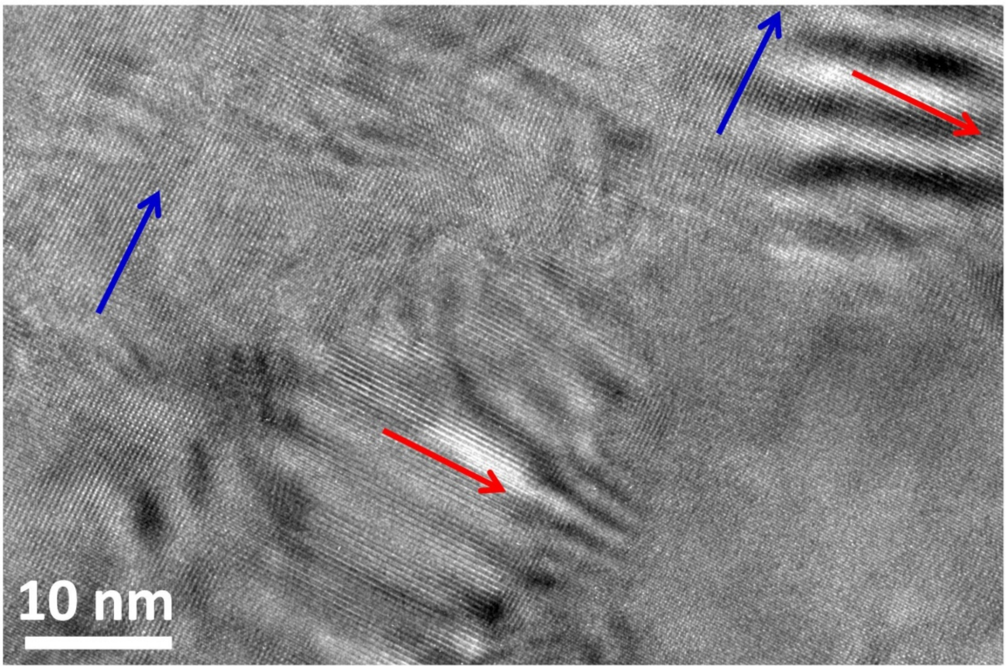


**Figure S2**. Plane-view transimission electron microscopy image of DMO/STO (001) film, in which the twin-like domain structure is illustrated.

In the above transimission electron microscopy image, a twin-like domain structure can be seen clearly, and the domain size is around 20-30 nm which is much larger than the period of the SSO which is just several unit cells.

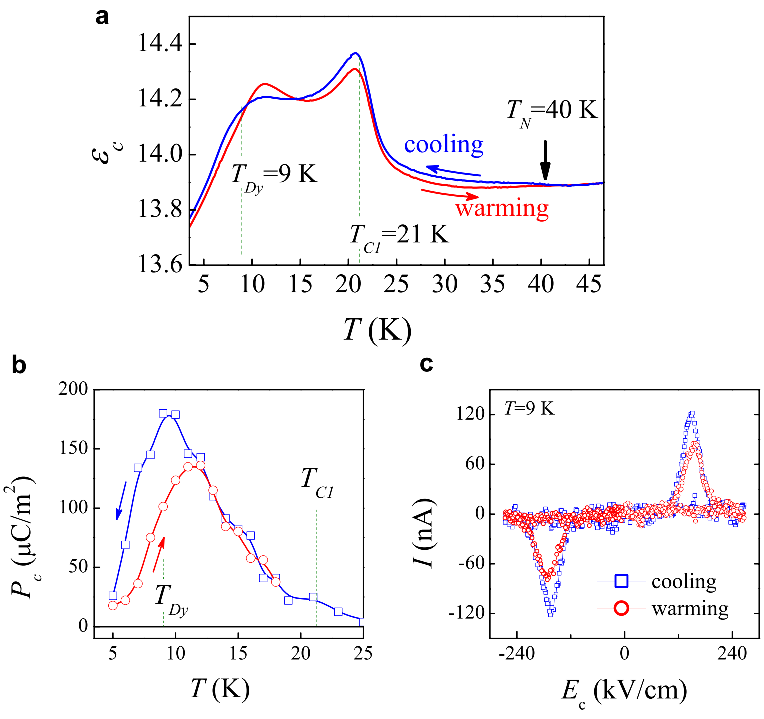


**Figure S3**. Multiferroic properties in $DyMnO_3$ thin film. (**a)** Dielectric constant as a function of temperature measured during the cooling and heating processes, in which three phase transitions can be indicated. (**b)** Polarization as a function of temperature measured using PUND during cooling and heating. (**c)** The inset shows the electric field dependence of ferroelectric switching current at 9 K, which were collected after cooling the sample from the paramagnetic phase (blue open squares), and then heating up the sample from 2 K (red circles), respectively. Clear difference between the two curves can be seen.

In Figure S3a are presented two $\varepsilon_c(T)$ curves measured in the cooling-warming cycle under $H$ = 0. In spite of slight difference, their general features are similar: three clear anomalies at $T_N$ = 40 K, $T_{C1}$ = 21 K, and $T_{Dy}$ = 9 K, arising respectively from the sinusoidal AFM ordering and *bc*-spiral spin ordering of Mn spins, as well as the CM ordering of Dy spins [23], suggesting the similar physics underlying the present DMO thin films with bulk DMO. The weak dielectric hysteresis reflects the thermal history dependence of the spin

structure. However, one can't find substantial feature for the coherent Dy spin ordering with the Mn spiral spin order at $T_{C2}$, similar to the case of bulk DMO.

The PUND method measured $P_c$-$T$ curves in the similar thermal cycle are plotted in Figure S3b. The $P_c$ emerges below $T_{C1}$ and increases quickly with decreasing $T$, due to the dual magnetic nature of the polarization. At $T_{\mathrm{Dy}}$, the $P_c$ reaches the maximal roughly at ~ $T_{\mathrm{Dy}}$ and then falls down gradually due to the ICM-CM transition of Dy spins, disabling the Dy-Mn exchange striction [20]. This transition is somehow of first order with thermal hysteresis [20], which leads to the same thermal history of the $P_c(T)$. The distinct difference in polarization current ($I$) as a function of electric field $E$ at $T$ = 9 K between the cooling and warming sequences is plotted in Figure S3c. Such thermal history characteristic may not be detectable by the pyroelectric current method usually run only in the warming process[24,36]. In this sense, the so far measured data basically reproduce the results of bulk DMO.

**References:**

35. Zhang, N., Dong, S., & Liu, J. –M., Ferroelectricity generated by spin-orbit and spin-lattice coupling in multiferroic $DyMnO_3$, *Front. Phys.* **7**, 408 (2012).

36 Goto, T., Kimura, T., Lawes, G., Ramirez, A. P., & Tokura, Y., Ferroelectricity and giant magnetoresistance in perovskite rare-earth manganites, *Phys. Rev. Lett.* **92**, 257201 (2004).
